# Importierte Inflation und Zinswende: Herausforderungen für die EZB

**DOI:** 10.1007/s10273-022-3206-4

**Published:** 2022-06-24

**Authors:** Überblick

**Keywords:** E31, E52, E58

## Abstract

Die Inflation in Deutschland und im gesamten Euroraum ist in den vergangenen Monaten stark gestiegen. Diese Entwicklung wird vor allem durch steigende Energiepreise getrieben. Die EZB hat angekündigt, das Anleihenkaufprogramm APP im 3. Quartal auslaufen zu lassen, und plant, den Leitzins noch diesen Sommer zu erhöhen. Sind die geplanten Maßnahmen ausreichend, um die Inflation zu senken? Es scheint wichtig, die Inflationserwartungen zu begrenzen, um Lohn-Preis-Spiralen zu verhindern. Auch ist zu fragen, wie wirksam Geldpolitik ist, wenn primär eine (importierte) Energiepreisinflation bekämpft werden muss. Zudem müssen die mit Zinserhöhungen verbundenen kontraktiven Effekte auf die Gesamtwirtschaft diskutiert werden, die durch Coronakrise und Ukrainekrieg ohnehin fragil ist.

## Abstract (en)

Inflation in Germany and the euro area as a whole has risen sharply in recent months. This development is mainly driven by rising energy prices. The ECB has announced that it will phase out the APP bond-buying programme in the third quarter and plans to raise the key interest rate this summer. Are the planned measures sufficient to lower inflation? It seems important to limit inflation expectations in order to prevent wage-price spirals. It also begs the question about how effective monetary policy is if primarily (imported) energy price inflation has to be fought. In addition, there must be a discussion about the contractionary effects of interest rate hikes on the economy as a whole, which is already fragile due to the coronavirus crisis and the Ukraine war.

